# Sensorless Control of Ultra-High-Speed PMSM via Improved PR and Adaptive Position Observer

**DOI:** 10.3390/s25051290

**Published:** 2025-02-20

**Authors:** Xiyue Bai, Weiguang Huang, Chuang Gao, Yingna Wu

**Affiliations:** 1 Center for Adaptive System Engineering, ShanghaiTech University, Shanghai 201210, China; baixy2022@shanghaitech.edu.cn (X.B.);; 2 Shanghai Advanced Research Insititute, Chinese Academy of Sciences, Shanghai 201210, China

**Keywords:** sensorless control, permanent magnet synchronous motor (PMSM), discrete model, proportional resonant (PR) controller, phase-locked loop (PLL)

## Abstract

To improve the precision of the position and speed estimation in ultra-high-speed (UHS) permanent magnet synchronous motors (PMSM) without position sensors, multiple refinements to the traditional extended electromotive force (EEMF) estimation algorithm are proposed in this paper. The key improvements include discretization compensation, high-frequency harmonic filtering, and the real-time adjustment of the phase-locked loop (PLL) bandwidth. Firstly, a discrete model is introduced to address EMF cross-coupling issues. Secondly, an improved proportional resonant (IPR) controller eliminating static errors is utilized in place of the conventional proportional-integral (PI) controller and low-pass filter (LPF) to enable precise electromotive force extraction, effectively filtering high-frequency harmonics that arise in low carrier ratio conditions. Based on a standard PR design, the IPR controller offers a streamlined calculation for target leading angles in delay compensation schemes to effectively mitigate discretization and delay errors. Additionally, an adaptive phase-locked loop (AQPLL) dynamically adjusts its bandwidth during acceleration to balance noise rejection and phase delay, reducing position estimation errors and optimizing torque. Simulations and experimental analyses on a motor (90,000 rpm, 30 kW) validate the effectiveness of the proposed sensorless driving techniques and demonstrate enhanced performance in position and velocity estimation, compared to the conventional EEMF approach.

## 1. Introduction

The PARstart permanent magnet synchronous motor (PMSM) is extensively utilized in various applications owing to its high power density, superior efficiency, and excellent control performance [1,2]. The PMSM used in this study can be directly connected to a gas turbine, effectively reducing the size of the gas turbine power generation system. However, the characteristics of ultra-high-speed motors, such as the high current frequency, low inductance, and high fundamental frequency, lead to issues like a large current ripple, high torque pulsation, and increased motor losses. Additionally, the drive system features a high fundamental frequency with a low carrier ratio, necessitating enhanced dynamic performance from the current loop [1]. For the high-performance control of a PMSM, accurate rotor position information is essential. However, mechanical position sensors, which depend on mechanical components to gauge position, struggle to match the swift changes encountered in an ultra-high-speed PMSM. Mechanical position sensors add friction and rotational inertia, are susceptible to damage from vibration and shock, require regular maintenance and calibration, and are unsuitable for high-speed, high-reliability, and low-maintenance environments [3,4].

Researchers have conducted extensive studies on sensorless drives. In medium- to high-speed motor applications, position estimation methods based on back electromotive force (EMF) have emerged prominently from a variety of rotor position estimation techniques [5,6]. EMF-based methods primarily include sliding mode observers (SMO) [7,8], state observers [9], model adaptive system (MRAS) estimators [10], Kalman filters [11], and disturbance observers [12,13]. Although these methods theoretically provide high estimation accuracy, they face challenges such as algorithmic complexity, implementation difficulty, difficulty in selecting regulator parameters, and strong dependence on motor parameters. These limitations become more pronounced during ultra-high-speed operation, where the sensitivity to regulator parameters increases, and motor parameters can vary significantly under high temperatures, restricting many of these approaches to laboratory research stages. For example, the sliding mode observer stands out as a prevalent method for medium- to high-speed sensorless control, favored for its robust disturbance rejection, effective dynamic performance, and minimal sensitivity to parameter variations. However, at low carrier ratios, an increased control delay decreases the effective switching frequency of the sign function, exacerbating the chattering in sliding mode observers and impairing the filtering efficacy of the low-pass filter (LPF). Therefore, after trying algorithms such as the sliding mode control and Kalman filtering, this work chose the extended back-electromotive force method EEMF, which has strong parameter robustness, no chattering problems, and a low computational burden.

The extended electromotive force (EEMF) estimation method [14] utilizes the motor armature current and voltage to calculate flux linkage and speed information. This method is straightforward and easy to implement, making it widely used in practical engineering for speed and position identification during medium-to-high-speed operations. For high-speed sensorless drives, due to the limitations of inverter switching frequency, the sampling frequency $f_{\mathrm{sample}}$ and rotor operating frequency $f_{e}$ ratio is typically low. Under low carrier ratio conditions, the resolution of the current and voltage signals decreases, affecting the accuracy of the EMF estimation in the EEMF method, which directly impacts the position and speed precision [15,16]. If the effects of digital interfaces, such as zero-order hold (ZOH), are overlooked in the EMF estimation at low $f_{\mathrm{sample}}/f_{e}$, it can lead to transient oscillations and issues with system stability. Under these conditions, continuous-time EMF estimations, as developed in [17], may prove inadequate for high-speed sensorless drives.

Due to factors such as inverter nonlinearity and the non-ideal distribution of stator windings, the estimated back EMF voltage may contain high-order harmonics [18]. The EEMF method typically relies on filters to extract the back EMF signal, such as adaptive notch filters [19], second-order generalized integrators [20,21], and filters based on adaptive linear neural networks [22]. However, under low carrier ratios, introducing filters also increases control delay, degrades filtering performance, and consequently affects the system control performance. Proportional resonant (PR) controllers can generate resonance at the fundamental frequency of the input current, achieving high gain. PR controllers track sinusoidal reference signals more effectively, eliminate steady-state errors, and improve dynamic characteristics, particularly during DC voltage dips or sudden load changes [4]. In contrast, PI controllers may exhibit steady-state errors when tracking sinusoidal signals.

The EEMF method typically encounters issues with suboptimal dynamic performance when observing rotor position over a wide speed range. Specifically, the rotor position error cannot adaptively diminish, resulting in a constant steady-state error. Sensorless position estimation commonly employs the quadrature phase-locked loop (QPLL) to monitor motor angular velocity and rotor position [23]. Although the EEMF method demonstrates robust parameter tolerance, it still introduces hysteresis error in position estimation, necessitating compensation for improved accuracy. The effectiveness of the QPLL depends on achieving a balance between rapid rotor tracking and phase delay minimization, which is influenced by the suppression of input noise [24]. However, a fixed QPLL bandwidth poses limitations for ultra-high-speed motors. In particular, during variations in the reference speed, tracking delays in the rotor estimation process can lead to insufficient decoupling in field-oriented control (FOC), resulting in torque availability losses. This degrades rotor dynamic control and diminishes overall motor performance. Recent years have seen efforts to enhance PLL through gradient descent optimization [25] or by designing adaptive controllers [26] to achieve improved robustness under speed disturbances.

To enhance the dynamic performance of ultra-high-speed motor position observers at low carrier ratios, this paper proposes the following improvements to the traditional EEMF method:

- We developed a discretized model of the conventional EEMF method and performed EMF decoupling under ultra-high-speed conditions.
- We substituted the PI controller in the EEMF method with an improved PR approach to eliminate harmonics during ultra-high-speed motor operation and eliminate static error.
- We added a quadrature phase-locked loop with adaptive bandwidth (AQPLL) to replace the traditional QPLL, enabling real-time bandwidth adjustment to enhance the dynamic tracking of rotor position, thereby maximizing the motor acceleration capability.
- The improved algorithm was embedded in a DSP, and an experimental platform was built using a 90,000 rpm and 30 kW motor to confirm the efficacy of all the suggested sensorless drive techniques.

## 2. Extended EMF Method

### 2.1. Principle of Extended Back EMF Method

**Figure 1:**
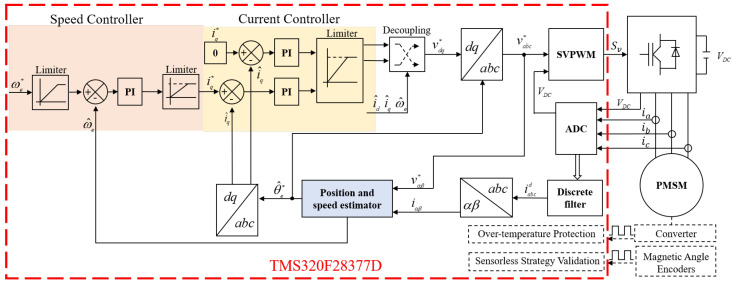
Overview of DSP-based sensorless FOC strategy.

**Figure 2:**
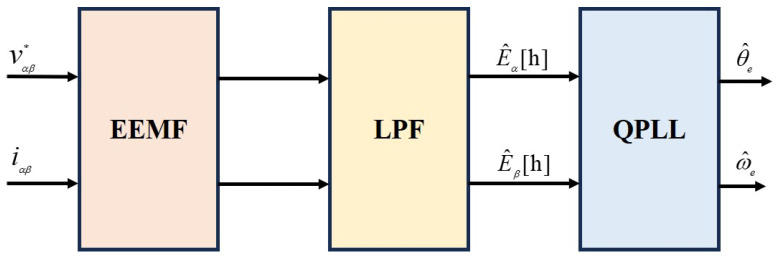
Overview of the conventional sensorless control strategy.

Figure 1 presents the field-oriented control (FOC) of the sensorless surface-mounted permanent magnet synchronous motor (SPMSM) drive, utilizing the extended electromotive force (EEMF) observer, which also serves as a simplified model for the DSP control panel utilized in the experimental setup detailed in Section 5. The position and speed estimation module, highlighted in blue, incorporates internal submodules (as shown in Figure 2), including the EEMF observer, filter, and PLL. In the experiments, the direct-axis current was set to zero, and flux-weakening control was applied under specific conditions.

The proposed method for position and speed estimation employs an extended electromotive force (EEMF)-based controller to estimate the back electromotive force (EMF) within the αβ reference frame. To mitigate high-speed-induced higher-order harmonics, this estimated back EMF undergoes low-pass filtering. Finally, the filtered EMF signal is processed through the PLL to accurately determine the rotor’s position and speed.

The continuous-domain extended back electromotive force (EMF) model of a surface permanent magnet synchronous motor (SPMSM) in the αβ coordinate system is given by

(1) $$\left[\begin{array}{c} u_{\alpha }\\ u_{\beta } \end{array}\right]=\left[\begin{array}{cc} R+pL_{d} & 0\\ 0 & R+pL_{d} \end{array}\right]\left[\begin{array}{c} i_{\alpha }\\ i_{\beta } \end{array}\right]+\left[\begin{array}{c} E_{\alpha }\\ E_{\beta } \end{array}\right].$$

In the provided equation, $u_{\alpha }$ and $u_{\beta }$ signify the voltages, while $i_{\alpha }$ and $i_{\beta }$ represent the currents along the αβ axes. The terms $E_{\alpha }$ and $E_{\beta }$ denote the back electromotive forces (EMFs) in the corresponding axes. The symbols *R* and $L_{d}$ are used to indicate the stator resistance and direct-axis inductance. Furthermore, *p* is utilized to denote the differential operator.

The back EMF can be expressed as

(2) $$E=\left[\begin{array}{c} E_{\alpha }\\ E_{\beta } \end{array}\right]=\omega \psi_{f}\left[\begin{array}{c} -\sin\theta \\ \cos\theta \end{array}\right].$$

In this context, ω denotes the electrical angular speed of the rotor, $\psi_{f}$ represents the rotor flux linkage, and θ indicates the rotor’s position. Analysis of Equation (2) reveals that the extended components of the back electromotive force incorporate information about the rotor’s position. By implementing an observer, it is feasible to estimate the extended back EMF, which allows for the accurate determination of both the rotor’s position and speed.

In Equation (1), $u_{\alpha }$, $u_{\beta }$, $i_{\alpha }$, and $i_{\beta }$ are directly measurable and are utilized as inputs for the observer. While the extended back electromotive force (EMF) components are not directly measurable, an estimate can be obtained by calculating the discrepancy between the measured and estimated currents and processing this difference through a PI controller.

The estimated currents, denoted as ${\hat{i}}_{\alpha }$ and ${\hat{i}}_{\beta }$, are computed as follows;

(3) $$\left\{\begin{array}{c} \left(pL_{d}+R\right){\hat{i}}_{\alpha }=u_{\alpha }+\frac{k_{p}s+k_{i}}{s}\left(i_{\alpha }-{\hat{i}}_{\alpha }\right)\\ \left(pL_{d}+R\right){\hat{i}}_{\beta }=u_{\beta }+\frac{k_{p}s+k_{i}}{s}\left(i_{\beta }-{\hat{i}}_{\beta }\right) \end{array}\right..$$

**Figure 3:**
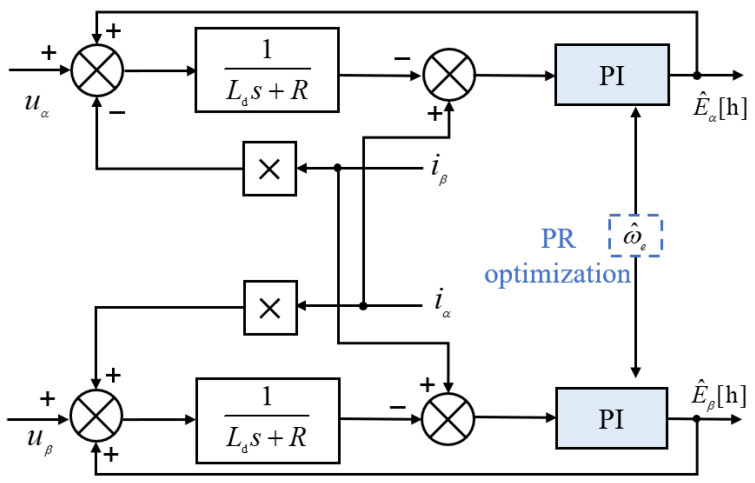
Conventional EEMF observer.

In the analysis, $k_{p}$ and $k_{i}$ are the proportional and integral gains, respectively. The estimated currents, ${\hat{i}}_{\alpha }$ and ${\hat{i}}_{\beta }$, represent the current components along the α and β axes, respectively. The configuration of the extended back EMF observer is illustrated in Figure 3.

### 2.2. Discrete Extended Back EMF Model

In practical applications, control algorithms are typically implemented on digital controllers, necessitating the discretization of observers originally formulated in the continuous domain. Discretizing the continuous model not only introduces discretization errors but also inadequately captures the cross-coupling effects between back EMF components, thereby impacting the accuracy of back EMF estimation [27]. To mitigate these issues, reconstructing the extended back EMF model in the discrete domain is essential.

**Figure 4:**
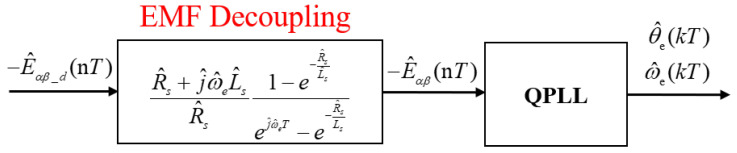
EMF cross-coupling decoupling during A/D conversion.

To develop the discrete-time model, the differential equations from the continuous-time extended back EMF expression were transformed (1) and are solved by substituting the time variable with the sampling variable nT, the EMF cross-coupling decoupling during A/D conversion model is shown in Figure 4. The detailed derivation process is outlined in [28], yielding the discrete-time expression for $I_{\alpha \beta }\left(t\right)$ in the stationary reference frame as follows:

(4) $$\begin{array}{c} I_{\alpha \beta }\left(nT\right)=e^{-\frac{R_{s}}{L_{s}}T}I_{\alpha \beta }\left[\left(n-1\right)T\right]+\frac{1-e^{-\frac{R_{s}}{L_{s}}}}{R_{s}}V_{\alpha \beta }\left[\left(n-1\right)T\right]-\frac{e^{j\omega_{e}T}-e^{-\frac{R_{s}}{L_{s}}T}}{R_{s}+j\omega_{e}L_{s}}E_{\alpha \beta }\left[\left(n-1\right)T\right]. \end{array}$$

When $\frac{f_{\mathrm{pwm}}}{f_{e}}$ is sufficiently low, the EMF obtained via the zero-order hold deviates from the sampled EMF due to cross-coupling effects. This results in a discrepancy between the discretized EMF $E_{\alpha \beta_{d}}\left(kT\right)$ and the sampled EMF $E_{\alpha \beta }\left(nT\right)$.

(5) $$E_{\alpha \beta_{-}d}\left(nT\right)=\left[\frac{R_{s}}{R_{s}+j\omega_{e}L_{s}}\frac{e^{j\omega_{e}T}-e^{-\frac{R_{s}}{L_{s}}}}{1-e^{-\frac{R_{s}}{L_{s}}}}\right]E_{\alpha \beta }\left(nT\right).$$

From the above equation, it is known that at low speeds, when $\omega_{e}$ approaches zero, the discrepancy between the sampled EMF $E_{\alpha \beta }\left(nT\right)$ and the discretized EMF $E_{\alpha \beta_{-}d}\left(nT\right)$ can be neglected. As the speed increases, the discrepancy between $E_{\alpha \beta_{-}d}\left(nT\right)$ and $E_{\alpha \beta }\left(nT\right)$ also gradually increases, and the performance of the sensorless drive will decrease accordingly.

### 2.3. PLL

**Figure 5:**
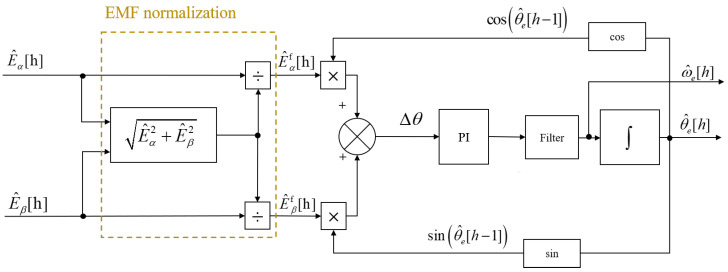
Conventional QPLL structure.

Subsequent to the observer, the quadrature phase-locked loop (QPLL) is employed, as depicted in Figure 2. The QPLL facilitates the acquisition of estimates for the rotor’s electrical speed, denoted as ${\hat{\omega }}_{e}$ and position ${\hat{\theta }}_{e}$, derived from the estimated back EMF. It also offers filtering capabilities to enhance the signal integrity [29]. Typically, the estimated back EMF is directly relayed from the output of the observer to the input of the QPLL. However, in scenarios involving high rotational speeds, it is advisable to introduce a filter prior to PLL to mitigate harmonic distortions. The filtered EMF is then used as the input to the QPLL, with the resultant speed and position signals serving as the outputs. The configuration of the QPLL is shown in Figure 5.

To maintain a consistent bandwidth of the QPLL across all motor speeds, it is essential to normalize the input, as discussed in [22]. The normalization of the estimated back EMF is as follows:

(6) $${\hat{e}}_{n\alpha \beta }={\hat{e}}_{\alpha \beta }/\left(\sqrt{{\hat{e}}_{\alpha }^{2}+{\hat{e}}_{\beta }^{2}}\right).$$

As shown in Figure 5, the discrepancy between the estimated angle and the actual angle is characterized as follows:

(7) $$\Delta \theta ={\hat{E}}_{\alpha }^{f}\cos\hat{\theta }+{\hat{E}}_{\beta }^{f}\sin\hat{\theta }\approx A\left(\theta -{\hat{\theta }}_{e}\right).$$

Here, the normalized vector of the estimated back EMF is denoted as ${\hat{E}}_{\alpha \beta }^{f}$. After normalization, the bandwidth of the QPLL becomes independent of the rotor speed, and its transfer function is specified as follows [30]:

(8) $$G_{PLL}\left(s\right)=\frac{k_{p}s+k_{i}}{s^{2}+k_{p}s+k_{i}}.$$

The bandwidth of the observer significantly influences the accuracy of the EEMF. A larger observer bandwidth introduces substantial noise into the estimated back EMF signal, causing significant fluctuations in the estimated rotor position determined through the arctangent function. Therefore, choosing an appropriate QPLL bandwidth is critical for accurately estimating the rotor position and speed values in a sensorless observer.

### 2.4. LPF

Under conditions of low carrier ratios, the presence of high-frequency harmonics escalates, mandating the integration of a low-pass filter (LPF) to mitigate these components and diminish noise in position estimation, thereby safeguarding transient response [25]. Nonetheless, the discretization of the LPF introduces a phase delay in the output signal, consequently degrading the steady-state performance of PLL. Such configurations of the PLL and LPF struggle to track rapid speed variations accurately, culminating in inevitable errors in position and speed estimates during transient phases, particularly under abrupt load changes typical in power steering motor operations. Consequently, the initial rotor position estimates derived from the EEMF observer fail to simultaneously accommodate both steady-state fidelity and transient responsiveness.

## 3. PR

### Traditional Dual-Integrator Proportional Resonant Controller

According to the internal model principle, the proportional resonant (PR) controller [31] is capable of tracking alternating current (AC) signals with zero steady-state error. The adoption of dual integrators is a prevalent strategy to facilitate frequency adaptation while minimizing additional computational demands. Consequently, the PR controller can supersede the traditional PID controller, eliminating tracking discrepancies, achieving zero phase shift in the back electromotive force (EMF), and enhancing both the precision and stability margin of back EMF estimation.

However, these schemes introduce phase lead errors at the resonant frequency and in delay compensation during the discretization process. As described in [32,33], a PR controller for tracking the *h*-th harmonic can be represented in the *s*-domain as follows:

(9) $$G_{PR}=k_{p}+\frac{k_{r}s}{s^{2}+h^{2}\omega_{0}^{2}}.$$

Here, $k_{p}$ and $k_{r}$ are the proportional coefficient and resonant coefficient, respectively, and $\omega_{0}$ denotes the resonant frequency (fundamental frequency).

**Figure 6:**
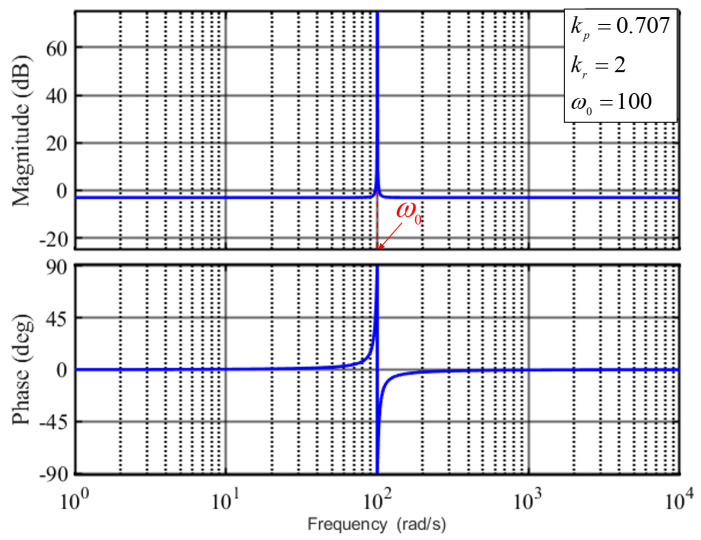
Bode diagram of conventional PR controller.

**Figure 7:**
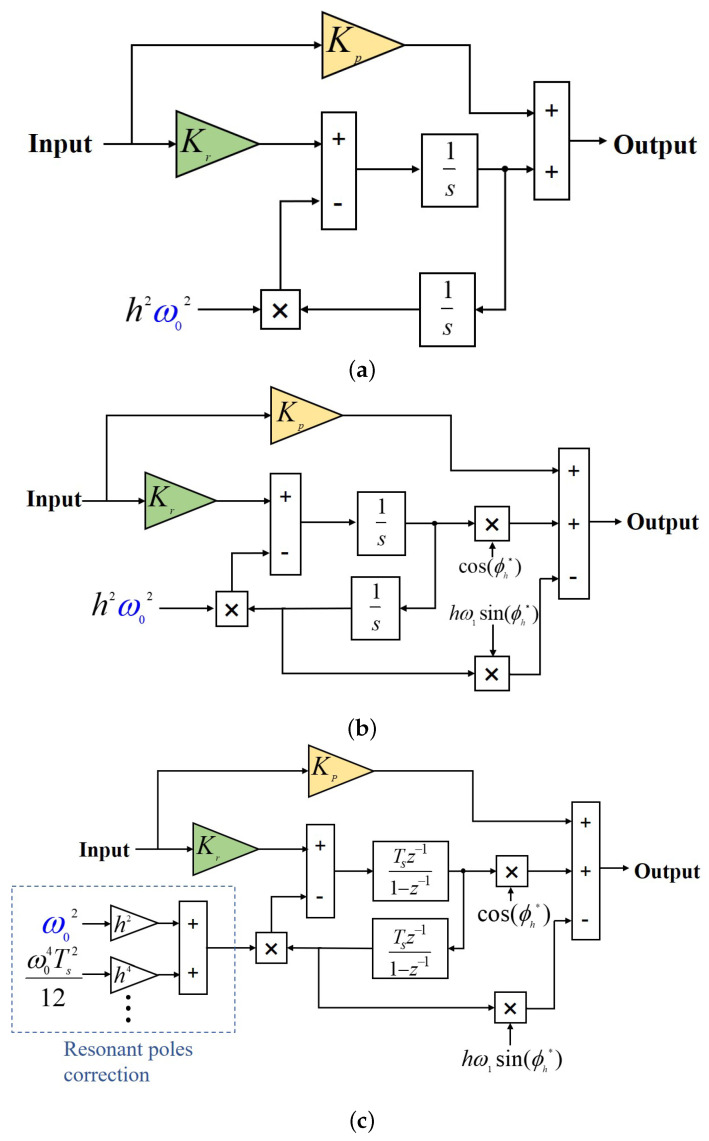
(**a**) Block diagrams of continuous-domain resonant controllers featuring dual integrators, denoted as $G_{PR_{h}}\left(s\right)$. (**b**) Resonant controllers with a phase lead $G_{PR_{h}}^{d}\left(s\right)$. (**c**) Digital resonant controllers with corrected poles $G_{PR_{h}}^{d}\left(z\right)$.

As shown in Figure 6, the Bode plot of the PR controller delineates its operational characteristics. Notably, the PR controller exhibits infinite gain at the resonant frequency $\omega_{0}$, enabling perfect tracking of signal components oscillating at frequency $h\omega_{0}$. Moreover, the gain decreases precipitously as the frequency moves away from the resonant point, which enables the PR controller to effectively suppress unwanted harmonics. This inherent filtering capability obviates the need for additional filtering components, thereby minimizing system delays.The schematic representation of the dual integrator configuration is depicted in Figure 7a.

To mitigate system delays—primarily arising from computational processes, modulation techniques, and passive filtering—a phase lead $\varphi_{h}$ is incorporated near the resonant frequency $h\omega_{0}$ of the resonant controller. The parameter $\varphi_{h}$ is quantified as the phase discrepancy between the actual phase response of the resonant controller and the ideal phase response provided by $G_{{\mathrm{PR}}_{h}}\left(s\right)$ at frequencies closely approximating $h\omega_{0}$. The *s*-domain formula for implementing this delay compensation in the PR controller is detailed in [34].

(10) $$G_{{\mathrm{PR}}_{h}}^{d}\left(s\right)=K_{P_{h}}+K_{I_{h}}\frac{s\cos\left(\phi_{h}^{*}\right)-h\omega_{0}\sin\left(\phi_{h}^{*}\right)}{s^{2}+h^{2}\omega_{0}^{2}}$$

In this context, $\phi_{h}^{*}$ represents the target phase lead angle, which is the intended value of $\phi_{h}$. In the continuous domain, $\phi_{h}$ is determined by the equation $\phi_{h}=$$\arctan\left(h\omega_{0}L_{d}/R\right)\neq 0$, while $\phi_{h}^{*}$ can be described as $\phi_{h}^{*}=\frac{\pi }{2}+\frac{3}{2}h\omega_{0}T_{s}$ [35]. Compared to the standard $G_{{\mathrm{PR}}_{h}}\left(s\right)$, the modified $G_{{\mathrm{PR}}_{h}}^{d}\left(s\right)$ improves system stability and diminishes irregular peaks within the closed-loop response.

Since the schemes depicted in Figure 7a,b are conceptualized in the continuous domain, direct implementation on digital devices is not feasible. The discretization process, essential for digital implementation, tends to diminish some of their key characteristics. Typically, the forward Euler method is employed to discretize the direct integrator, whereas the backward Euler method is utilized for the feedback integrator, as documented in [7,18]. This discretization approach results in the following transfer function in the *z*-domain:

(11) $$G_{PR_{h}}\left(z\right)=K_{P_{h}}+K_{I_{h}}T_{s}\frac{z^{-1}-z^{-2}}{1-2z^{-1}\left(1-h^{2}\omega_{0}^{2}T_{s}^{2}/2\right)+z^{-2}}$$

(12) $$\begin{array}{c} G_{PR_{\hbar }}^{d}=K_{P_{\hbar }}+K_{I_{h}}T_{S}\frac{z^{-1}\left[\cos\left(\phi_{\hbar }^{*}\right)-h\omega_{0}T_{s}\sin\left(\phi_{\hbar }^{*}\right)\right]-z^{-2}\cos\left(\phi_{\hbar }^{*}\right)}{1-2z^{-1}\left(1-h^{2}\omega_{0}^{2}T_{s}^{2}/2\right)+z^{-2}}. \end{array}$$

As demonstrated in [32], the discretization process weakens the key performance of the PR controller. Discretization causes a shift in the resonant pole of the controller, resulting in a deviation from the desired resonant frequency. This deviation can lead to significant steady-state errors.

To reduce the resonant frequency deviation caused by discretization, the position of the resonant pole can be corrected. Specifically, when designing the discretized model of the PR controller, the input signal can be expanded using a higher-order Taylor series to approximate the actual resonant frequency more closely. For example, increasing from a second-order to a fourth-order expansion provides better accuracy across most frequencies.

The improvement to Figure 7a is shown in Figure 7c.

(13) $$h^{2}\omega_{0}^{2}\to C_{h}=h^{2}\omega_{0}^{2}-h^{4}\frac{\omega_{0}^{4}T_{s}^{2}}{12}+h^{6}\frac{\omega_{0}^{6}T_{s}^{4}}{360}+\ldots$$

The above equation can be improved to

(14) $$\begin{array}{c} G_{PR_{h}}^{d}=K_{P_{h}}+K_{I_{h}}T_{s}\frac{z^{-1}\left[\cos\left(\phi_{h}^{*}\right)-h\omega_{0}T_{s}\sin\left(\phi_{\hbar }^{*}\right)\right]-z^{-2}\cos\left(\phi_{h}^{*}\right)}{1-2z^{-1}\left(1-C_{h}T_{s}^{2}/2\right)+z^{-2}} \end{array}.$$

## 4. AQPLL

### 4.1. Quadrature PLL with Adaptive Bandwidth

Unlike the operating frequency of high-speed PMSMs, which can reach several kilohertz, the fundamental voltage frequency of standard PMSMs typically ranges from tens to hundreds of Hz. To facilitate the QPLL design and adaptive filter convergence and to eliminate the impact of back EMF amplitude variation with speed, the traditional QPLL shown in Figure 5 uses a normalized back EMF design. This design simplifies the selection of constant poles and enables a uniform bandwidth for the QPLL across the full speed range, resulting in good disturbance rejection performance. However, in ultra-high-speed scenarios, the QPLL bandwidth must be sufficiently wide to cover the entire dynamic range of high-speed PMSMs.

**Figure 8:**
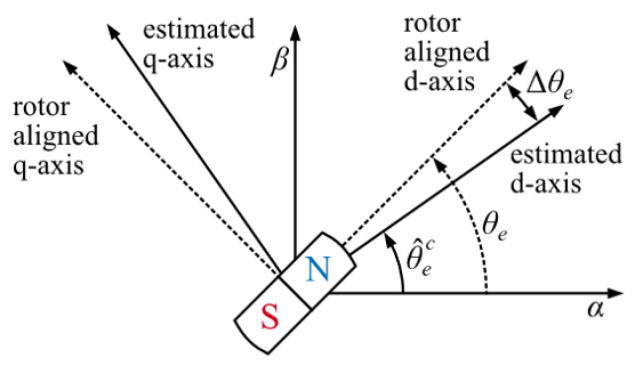
Rapid motor speed variations resulting from a low QPLL bandwidth lead to misalignment in the control frames.

Unfortunately, QPLL’s disturbance rejection and dynamic performance represent a trade-off: selecting a wider working bandwidth may compromise the QPLL’s disturbance rejection capability, while choosing a narrower bandwidth causes the QPLL to respond slowly to sudden changes in motor state (e.g., rotor load or speed variations) [23]. This can lead to position errors and even time misalignment in the control frame, as shown in Figure 8. During motor speed adjustments, significant discrepancies may arise between the estimated and actual dq currents, deteriorating current decoupling and potentially leading to motor torque and axial stability degradation. Since the motor used in this experiment is a gas turbine-embedded motor, axial stability degradation increases the risk of equipment faults and failures.

**Figure 9:**
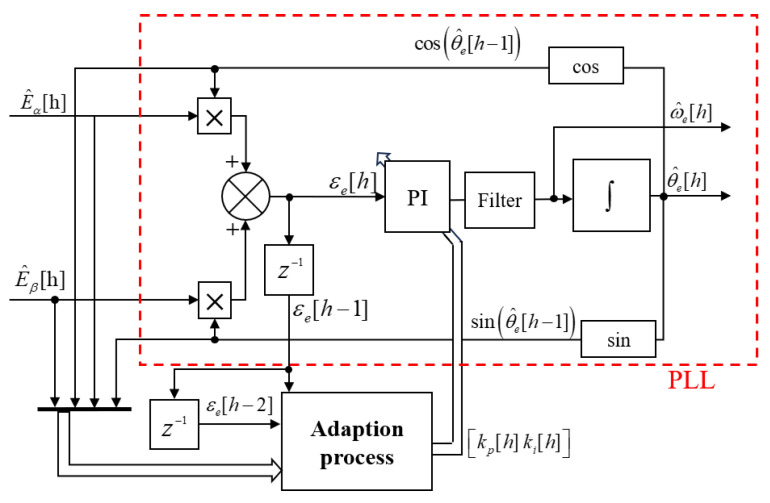
Block diagram of the adaptive QPLL.

To overcome these challenges, a quadrature phase-locked loop with adaptive bandwidth was designed, as illustrated in Figure 9. This method dynamically modifies its bandwidth, enabling quick responses to changes in motor operation. The adaptive strategy utilizes stochastic gradient descent to minimize the square error of the controller’s input signal by adaptively tuning the gains $k_{p}$ and $k_{i}$, thereby altering the poles as needed. The pole in Equation (8) can be substituted with the following expression:

(15) $$k_{p}\left[h\right]=2\zeta \eta \left[h\right],\;k_{i}\left[h\right]=\eta^{2}\left[h\right].$$

In this context, ζ represents the damping factor, and η denotes the natural frequency; together, these are referred to as the tuning parameters of the AQPLL. During the discretization process, a discrete time-step variable *h* is utilized to define specific time intervals. To prevent instability resulting from the parameter coupling effects, it is generally inadvisable to adjust both ζ and η simultaneously. Therefore, the primary tuning parameter η is selected for adjustment while keeping the damping factor ζ constant.

The parameters are adjusted as

(16) $$\eta \left[h\right]=\eta \left[h-1\right]-\mu \frac{1}{2}\left(\frac{\partial \varepsilon_{r}^{2}\left[h\right]}{\partial \eta [h-1]}\right).$$

In this context, μ serves as the critical step size parameter that governs the rate of adaptation, while $\varepsilon_{r}$ represents the input error to PI controller within the QPLL. The definitive formula for updating the tuning parameter is expressed as follows:

(17) $$\eta \left[h\right]=\eta \left[h-1\right]-\mu z_{1}\left[h\right]z_{2}\left[h\right]=\eta \left[h-1\right]-\Delta \eta \left[h\right].$$

Here, $z_{1}$ and $z_{2}$ are partial solutions to Equation (17); the detailed derivation process is described in [36], and they are expressed as

(18) $$\begin{array}{c} z_{1}\left[h\right]={\hat{E}}_{\alpha }^{f}\left[h\right]\sin\left({\hat{\theta }}_{e}\left[h-1\right]\right)-{\hat{E}}_{\beta }^{f}\left[h\right]\cos\left({\hat{\theta }}_{e}\left[h-1\right]\right)\\ z_{2}\left[h\right]=2\zeta \varepsilon_{r}\left[h-1\right]+T_{s}\eta \left[h-1\right]\left(\varepsilon_{r}\left[h-1\right]+\varepsilon_{r}\left[h-2\right]\right). \end{array}$$

In the equations, $T_{s}$ is the sampling time, and $\left[{\hat{E}}_{\alpha }^{f},{\hat{E}}_{\beta }^{f}\right]$ represents the output of the normalized back EMF (as shown in Figure 5). The parameter Δη[h] denotes the positive and negative step size increments.

### 4.2. Stability Analysis

Substitute the terms from Equation (15) into Equation (7).

(19) $$G_{PLL}\left(s\right)=\frac{2\varsigma \eta s+\eta^{2}}{s^{2}+2\varsigma \eta s+\eta^{2}}$$

The roots of the characteristic equation derived from the AQPLL transfer function can be determined and utilized to analyze the system’s stability with respect to variations in η.

(20) $$s_{1,2}=-\zeta \eta \pm \eta \sqrt{\left(\zeta^{2}-1\right)}$$

**Figure 10:**
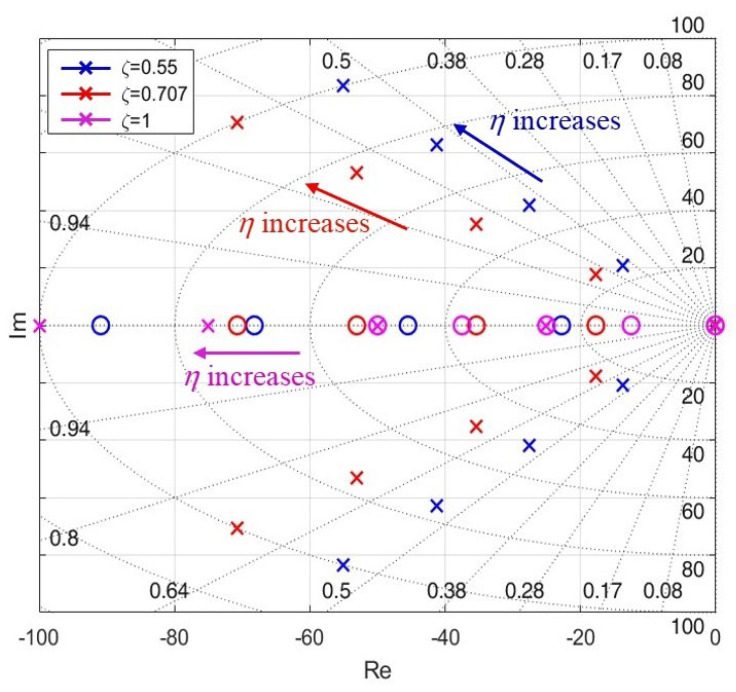
Pole-zero map of AQPLL with fixed damping factor and increased natural frequency.

Figure 10 displays the shifts in zeros and poles as η increases. It is observed that, provided η remains positive during the estimation process, the roots of the characteristic equation stay within the left half of the *s*-plane, confirming the stability of the AQPLL system. To circumvent potential challenges in practical scenarios, particularly at very low speeds where back EMF observation becomes challenging, the value of η may be further restricted.

(21) $$\eta \geq \eta_{\min}$$

Here, $\eta_{\min}$ represents the minimum (initial) value of η necessary to successfully lock onto the motor frequency during startup.

The state error of the observer will gradually decay under asymptotic stability conditions without divergence, ensuring accurate tracking of the system state and long-term stability. Given that the electrical time constant is notably smaller than the mechanical time constant in this system, it is reasonable to assume that the rate of change of the electrical angular frequency is essentially zero, ${\dot{\omega }}_{e}=0$. The observer’s bandwidth can be described by the difference between the estimated back EMF spatial vector $E_{\alpha \beta }$ and actual back EMF spatial vector $E_{\alpha \beta }$, whose complex form is presented in [27].

(22) $$\frac{{\hat{E}}_{\alpha \beta }}{E_{\alpha \beta }}=\frac{-K_{2}}{\left(s-j{\hat{\omega }}_{e}\right)\left(sL_{s}+R_{s}+K_{1}L_{s}\right)-K_{2}}$$

The selection of the observer gain needs to ensure that the observer can quickly adjust and maintain high-precision tracking of motor states (such as speed, position, and load disturbances) as they change. The design of the state feedback loop is closely related to system noise, observer bandwidth, and its dynamic characteristics.

(23) $${\hat{x}}_{\alpha \beta }=\left(A\left({\hat{\omega }}_{e}\right)-KC\right){\hat{x}}_{\alpha \beta }+Bv_{\alpha \beta }^{*}+K{\tilde{i}}_{\alpha \beta }$$

In the given equation, the estimated speed is incorporated into the observer state matrix, with *C* representing the observer output matrix, defined as $C=\left[\begin{array}{cc} 1 & 0\\ 0 & 1 \end{array}\right]$. Thus, the observer gain selected is effective across the entire range of motor speeds, ensuring that the closed-loop poles of Equation (19) are positioned in the left half of the *s*-plane. Crucially, the discretization process must maintain the stability as originally designed.

### 4.3. Phase Compensation

**Figure 11:**
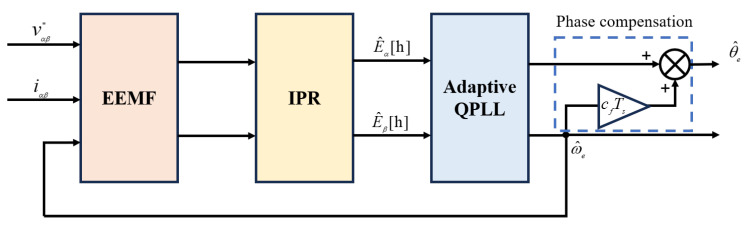
Overview of the optimization sensorless control strategy.

The enhanced modules for motor position and speed observation, building upon the setup shown in Figure 2, are depicted in Figure 11. The final module in Figure 11, designated as the phase compensation, addresses additional errors potentially introduced by variations in motor parameters (such as $L_{s}$ and $R_{s}$) due to application frequency, parameter saturation, and delays associated with digital and hardware components [37]. At nominal speed, the compensation factor $c_{f}$ is fine-tuned to minimize the phase current, ensuring that the estimated currents in the dq coordinate system are effectively decoupled and maximum torque is achieved. Given that the main emphasis of this study is on refining PR and AQPLL methods, $c_{f}$ is maintained as a constant, compensating solely for linear digital delays, which has proven adequate for achieving satisfactory performance in the motors utilized during the experimental evaluations.

## 5. Simulation and Experimental Results

**Figure 12:**
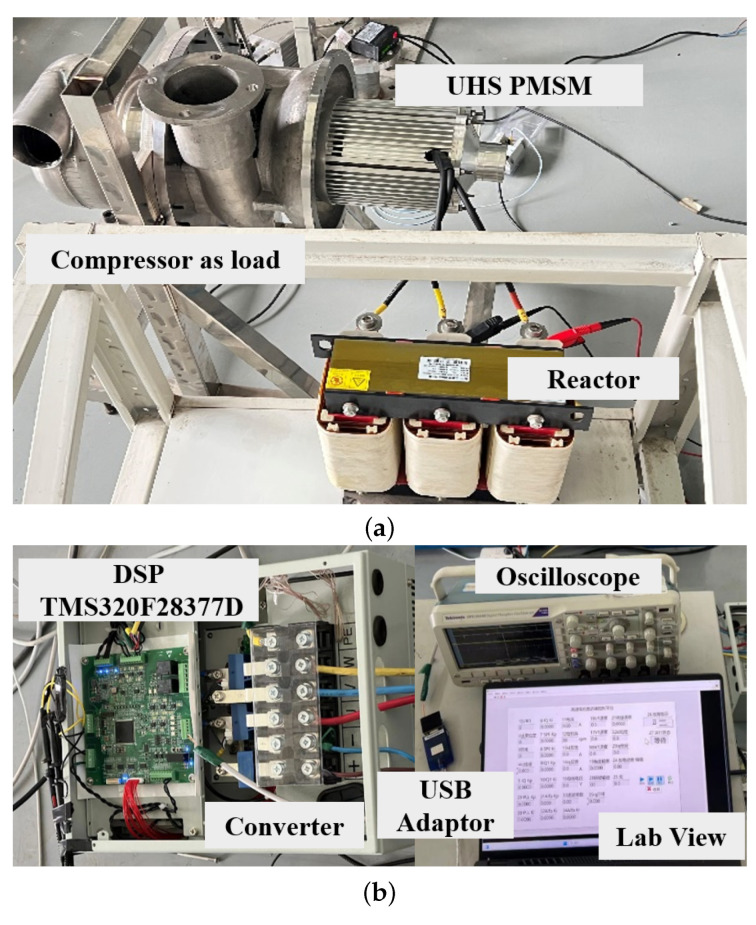
Experimental setup for sensorless FOC of the high-speed PMSM (**a**) Motor and compressor load. (**b**) Motor controller.

To assess the efficacy of the newly proposed IPR-based extended back EMF observer and AQPLL, the control strategy was integrated into a DSP and evaluated using a 30 kW PMSM drive system. The experimental setup is depicted in Figure 12, with Figure 12a illustrating the ultra-high-speed PMSM, its compressor load, and the reactor; Figure 12b showcases the controller, the converter, and the input/output connections, as well as the link between the controller and the host computer. The specifications of the PMSM drive system are detailed in Table 1. Current measurements were conducted using Hall effect current sensors, and the actual motor position and speed were determined using an internal magnetic encoder within the motor. In this paper, motor experiments were conducted under no-load conditions, and MATLAB 2022a simulations were performed under load conditions. Experiments under load conditions were not undertaken because the gas turbine compressor, used as the load, is unable to impose sudden load increases on the motor. The experimental categories and their respective results are outlined below.

### 5.1. Comparison of the Acceleration Performance Between Improved and Traditional Methods at Low Carrier Ratios

When the motor was not connected to the compressor, i.e., when it operated under no-load conditions, comparative experiments were conducted on three different methods: the traditional EEMF method, the proposed IPR-based extended back EMF observer and the AQPLL-modified phase-locked loop, with a switching frequency of 20 kHz.

**Figure 13:**
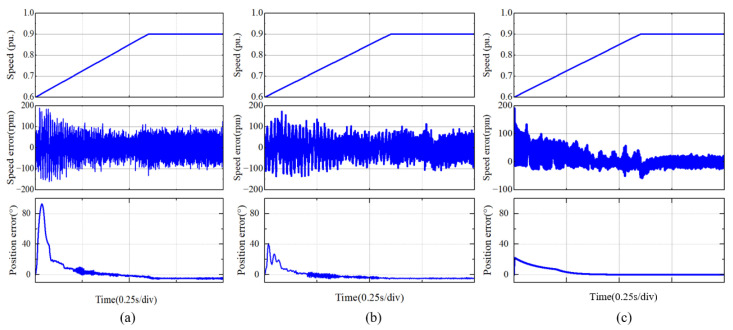
Comparison of experimental results corresponding to the time when the motor speed increases from 50,000 rpm to 90,000 rpm within 0.6 s: (**a**) traditional EEMF, (**b**) IPR-based EEMF, and (**c**) adding AQPLL.

Initially, a motor acceleration performance test was performed, where the motor speed was progressively increased from 50,000 rpm to 90,000 rpm within a span of 0.6 s. The test employed a switching frequency of 20 kHz, with carrier ratios set at 24 and 12.5. The position and angle errors as estimated by the conventional EEMF method are illustrated in Figure 13a. The errors using the IPR-based EEMF method are depicted in Figure 13b. Furthermore, the results for position and speed estimation enhanced by the addition of the AQPLL are presented in Figure 13c.

Comparing Figure 13a,b, it can be observed that the speed estimation filtered by the IPR results in a significant reduction in the overall speed error compared to the traditional method, with the average error decreasing from approximately 80 rpm to around 60 rpm. The position error during rapid response decreases from 80° to about 40°, and the amplitude of the position error during the recovery period is also reduced compared to the traditional method.

The comparison between Figure 13a,c demonstrates that the initial speed step change significantly enhances the motor speed estimation, reducing the impact of errors by nearly half. Throughout the acceleration process, the average motor speed error decreases from around 80 rpm to approximately 25 rpm, a reduction of nearly 70%. Compared to the traditional method, the AQPLL method provides faster recovery of the motor position error. It takes 0.2 s for the position error to decrease below 10° using the traditional method, while the EEMF method with AQPLL achieves this in just 0.1 s. The final motor position error decreases from 3° to within 1°.

### 5.2. Comparison of Load Performance Between Improved and Traditional Methods at Low Carrier Ratios

**Figure 14:**
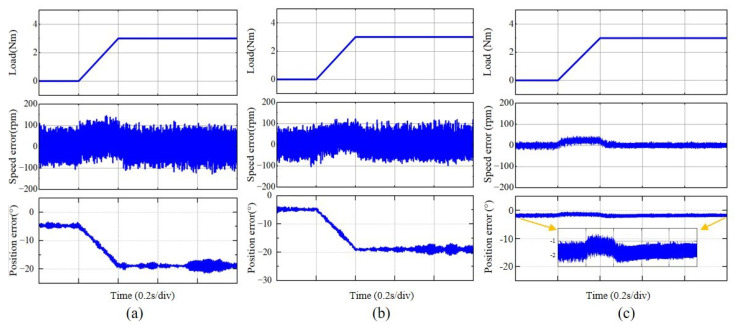
When the motor is running stably, a 3Nm load is added within 0.25s, and the corresponding change in speed and position is reached when the load reaches stable operation again: (**a**) traditional EEMF, (**b**) IPR-based EEMF, and (**c**) adding AQPLL.

Due to the fact that the motor load is the compressor section of a gas turbine, it is not feasible to achieve sudden load application. To directly observe the variations in motor speed and position before and after load application and to ensure optimal motor performance under load, this study employed MATLAB to simulate the motor’s response to a sudden load application. Figure 14 presents the experimental outcomes when a sudden load of 3 N·m (representing 80% of the maximum load capacity) was applied at 0.2 s, while the motor was operating at 90,000 rpm and a carrier ratio of 12.5. Comparing Figure 14a,b, it can be observed that, compared to the traditional EEMF method, the IPR method exhibits a smaller increase in the speed estimation error after load application, with significantly fewer harmonics. Additionally, the error rise is smoother when the motor experiences an impact, and the position error contains fewer harmonics.

Comparing Figure 14a,c, under sudden load conditions, the control method with AQPLL results in a smaller increase in the speed error. Notably, the position error in the AQPLL returns adaptively to its original error range once the load stabilizes. In contrast, the traditional EEMF method does not return to the original error range after the load stabilizes but instead maintains a position error of approximately 20°.

**Figure 15:**
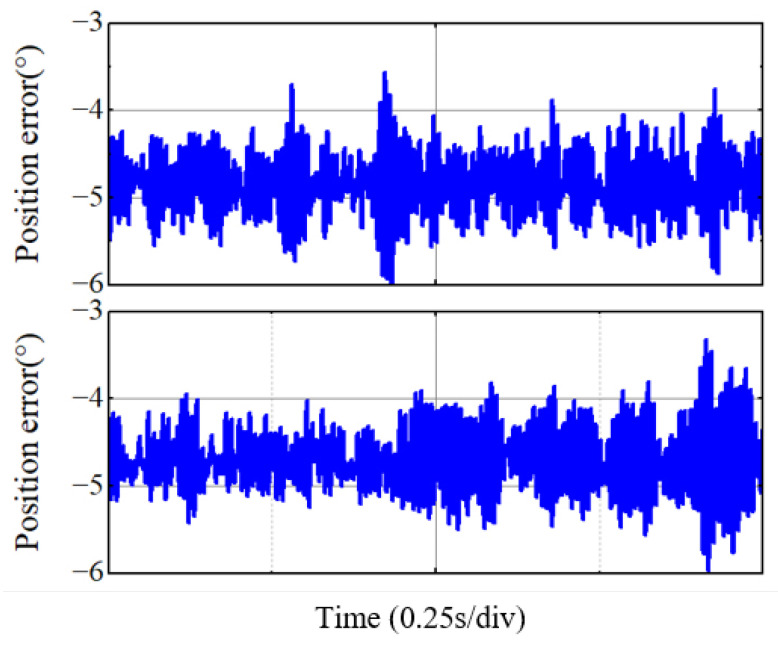
Three methods of position error when the motor reaches steady-state operation at 90,000 rpm.

Figure 15 illustrates the steady-state error in the motor position estimation. Comparing the two preceding figures, it is evident that the traditional EEMF method results in significant harmonics in the position estimation, making the motor more susceptible to axial displacement instability and thereby compromising the stability of the motor load. In contrast, the incorporation of the IPR algorithm effectively reduces the motor harmonics.

From the dynamic and steady-state experimental results, it is evident that the proposed IPR-based improved EEMF and AQPLL method exhibits excellent dynamic speed response and disturbance rejection performance under low carrier ratio conditions in ultra-high-speed motor applications at 90,000 rpm.

### 5.3. Local Characteristics of Motor Control Methods

**Figure 16:**
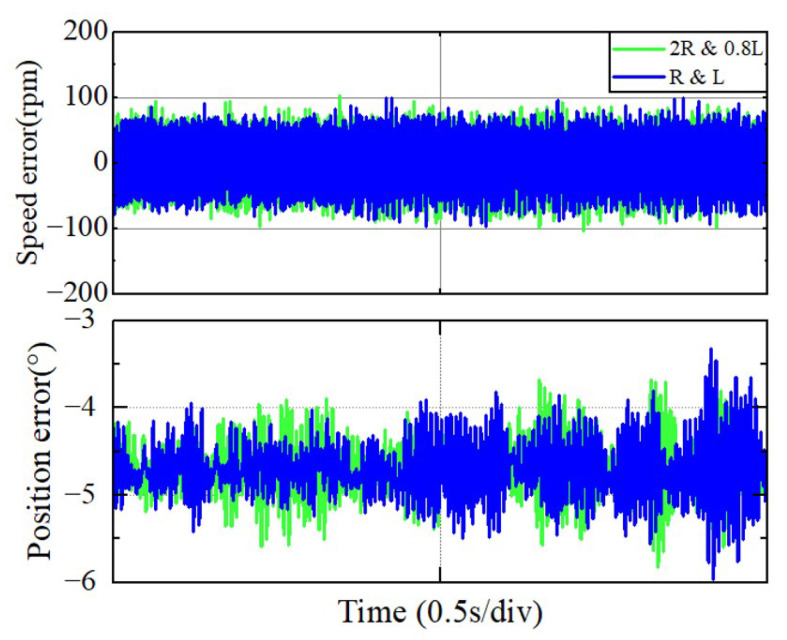
Speed and position estimation errors when the motor parameters change to 2R and 0.8L under the EEMF method.

As mentioned in Section 1 and Section 2, one of the primary reasons for selecting the EEMF method is its robustness to motor parameters, especially as the temperature rise during ultra-high-speed motor operation increases the resistance, and the skin effect reduces the motor’s equivalent inductance at high speeds. To assess the parameter robustness of the EEMF method, experiments were conducted comparing the steady-state performance of motor operation using altered parameters versus the original settings. The experimental findings are depicted in Figure 16. As indicated in Figure 16, changes in the parameters do not significantly impact the stability range of the position and speed estimation.

**Figure 17:**
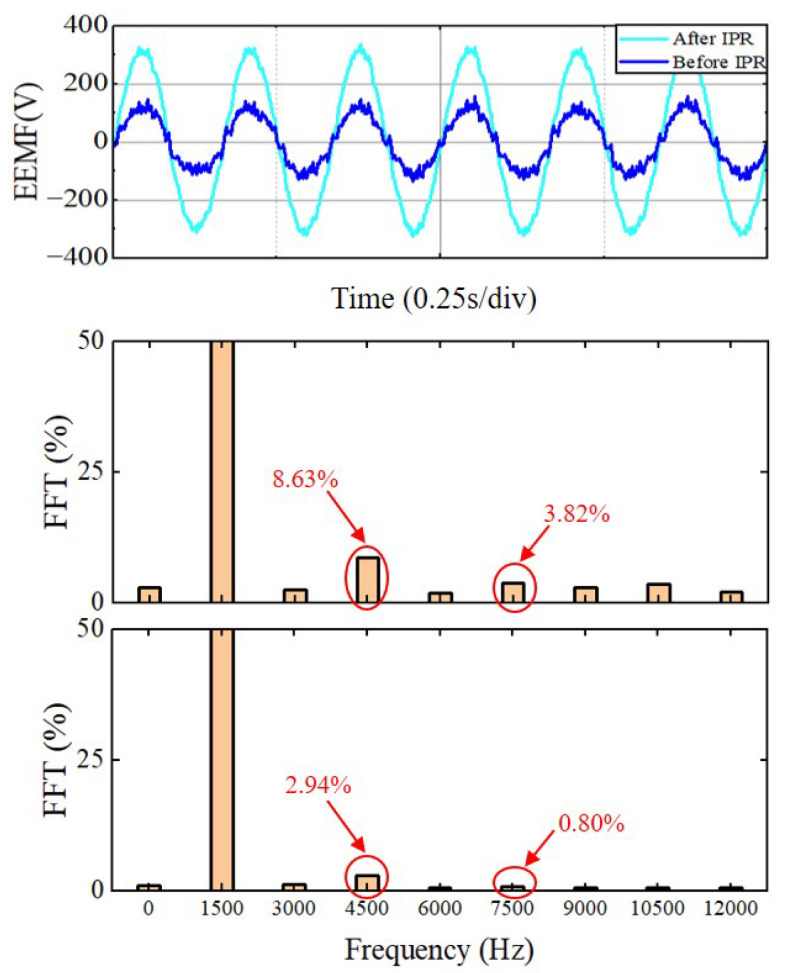
Comparison of harmonic frequencies of current and EMF signals before and after IPR.

To minimize harmonics in the estimated back EMF and alleviate the delays introduced by filters, the PI controller in EEMF setup was substituted with an IPR (improved proportional resonant) controller. The efficacy of the IPR controller was confirmed through the analysis of the final experimental results. As shown in Figure 17, the third and fifth harmonics in the FFT are significantly improved before and after the signal passes through the IPR, particularly with the third harmonic reduced from 8% to below 3%. The remaining harmonic frequencies are also improved overall, achieving steady-state error-free tracking and zero phase shift. Additionally, the IPR retains the original characteristics of the PI controller, providing large gains for current error.

In summary, the EEMF method demonstrates excellent robustness to motor parameter variations, replacing the PI and filter with the IPR effectively filters out harmonics, and the AQPLL method provides adaptive functionality for motor position and speed observation.

## 6. Conclusions

In this paper, we explore advancements in sensorless control for ultra-high-speed permanent magnet synchronous motors (UHS PMSM), targeting enhanced accuracy in position and speed estimation via an improved extended electromotive force (EEMF) estimation technique. Key contributions include the introduction of discretization compensation, high-frequency harmonic filtering, and the real-time adjustment of the PLL bandwidth. An enhanced proportional-resonant (IPR) controller is implemented to replace the traditional PI controller, effectively eliminating steady-state errors and improving harmonic filtering. Additionally, an adaptive quadrature PLL (AQPLL) is employed for dynamic bandwidth adjustment, optimizing motor performance during rapid acceleration.

The simulation and experimental results confirm that these methods substantially improve the estimation accuracy and disturbance rejection at operational speeds up to 90,000 rpm, thereby advancing both dynamic and steady-state motor control. Future research will focus on further refining these estimation techniques and implementing FPGA-based approaches to enhance the robustness and adaptability for real-time applications in advanced motor control systems.

## Figures and Tables

**Table 1:** Parameters of the PMSM drive system.

Parameter	Symbol	Value
DC Voltage	$U_{dc}$ (V)	800
Rated Power	$P_{N}$ (kW)	30
Rated Speed	$n_{N}$ (r/min)	90,000
Rated Torque	$T_{eN}$ (N·m)	3.75
Number of Pole Pairs	/	1
Rated Frequency	$f_{e}$ (Hz)	1500
Stator Resistance	*R* (Ω)	0.03
d/q-axis Inductance	$L_{d}/L_{q}$ (μH)	115
Permanent Magnet Flux	$\psi_{f}$ (Wb)	0.043
Moment of Inertia	*J* (kg·$m^{2}$)	$5.6188\times 10^{-5}$
Switching Frequency	$f_{\mathrm{sample}}$ (kHz)	20

## Data Availability

Due to commercial confidentiality reasons, the data cannot be made public.

## References

[1] Krähenbühl D., Zwyssig C., Weser H., Kolar J.W. (2010). A miniature 500,000-r/min electrically driven turbocompressor. IEEE Trans. Ind. Appl..

[2] Gerada D., Mebarki A., Brown N.L., Gerada C., Cavagnino A., Boglietti A. (2013). High-speed electrical machines: Technologies, trends, and developments. IEEE Trans. Ind. Electron..

[3] Tenconi A., Vaschetto S., Vigliani A. (2013). Electrical machines for high-speed applications: Design considerations and tradeoffs. IEEE Trans. Ind. Electron..

[4] Nicola M., Nicola C.I., Ionete C., Șendrescu D., Roman M. (2023). Improved Performance for PMSM Sensorless Control Based on Robust-Type Controller, ESO-Type Observer, Multiple Neural Networks, and RL-TD3 Agent. Sensors.

[5] Liang D., Li J., Qu R., Kong W. (2017). Adaptive second-order sliding-mode observer for PMSM sensorless control considering VSI nonlinearity. IEEE Trans. Power Electron..

[6] Ye T., Dai N., Lam C.S., Wong M.C., Guerrero J.M. (2016). Analysis, design, and implementation of a quasi-proportional-resonant controller for a multifunctional capacitive-coupling grid-connected inverter. IEEE Trans. Ind. Appl..

[7] Wang D., Liu X. (2024). Sensorless Control of PMSM with Improved Adaptive Super-Twisting Sliding Mode Observer and IST-QSG. IEEE Trans. Transp. Electrif..

[8] Zhang X., Li H., Shao M. (2024). Fixed-Time-Convergent Sliding Mode Control with Sliding Mode Observer for PMSM Speed Regulation. Sensors.

[9] Zhu G., Kaddouri A., Dessaint L.A., Akhrif O. (2001). A nonlinear state observer for the sensorless control of a permanent-magnet AC machine. IEEE Trans. Ind. Electron..

[10] Po-Ngam S., Sangwongwanich S. (2011). Stability and dynamic performance improvement of adaptive full-order observers for sensorless PMSM drive. IEEE Trans. Power Electron..

[11] Smidl V., Peroutka Z. (2011). Advantages of square-root extended Kalman filter for sensorless control of AC drives. IEEE Trans. Ind. Electron..

[12] Sun X., Cao J., Lei G., Guo Y., Zhu J. (2021). A robust deadbeat predictive controller with delay compensation based on composite sliding-mode observer for PMSMs. IEEE Trans. Power Electron..

[13] Deng Y., Zhu J., Liu H. (2023). The Improved Particle Swarm Optimization Method: An Efficient Parameter Tuning Method with the Tuning Parameters of a Dual-Motor Active Disturbance Rejection Controller. Sensors.

[14] Wang T., Huang J., Ye M., Chen J., Kong W., Kang M., Yu M. (2018). An EMF observer for PMSM sensorless drives adaptive to stator resistance and rotor flux linkage. IEEE J. Emerg. Sel. Top. Power Electron..

[15] Kim H., Degner M.W., Guerrero J.M., Briz F., Lorenz R.D. (2010). Discrete-time current regulator design for AC machine drives. IEEE Trans. Ind. Appl..

[16] Hinkkanen M., Awan H.A.A., Qu Z., Tuovinen T., Briz F. (2015). Current control for synchronous motor drives: Direct discrete-time pole-placement design. IEEE Trans. Ind. Appl..

[17] Yim J.S., Sul S.K., Bae B.H., Patel N.R., Hiti S. (2009). Modified current control schemes for high-performance permanent-magnet AC drives with low sampling to operating frequency ratio. IEEE Trans. Ind. Appl..

[18] Zhang G., Wang G., Xu D., Yu Y. (2017). Discrete-time low-frequency-ratio synchronous-frame full-order observer for position sensorless IPMSM drives. IEEE J. Emerg. Sel. Top. Power Electron..

[19] Wang G., Zhan H., Zhang G., Gui X., Xu D. (2013). Adaptive compensation method of position estimation harmonic error for EMF-based observer in sensorless IPMSM drives. IEEE Trans. Power Electron..

[20] Cheng H., Sun S., Zhou X., Shao D., Mi S., Hu Y. (2021). Sensorless DPCC of PMLSM using SOGI-PLL-based high-order SMO with cogging force feedforward compensation. IEEE Trans. Transp. Electrif..

[21] Jin R., Chen J., Hu P., Li J. (2024). Optimizing Sensorless Control in PMSM Based on the SOGIFO-X Flux Observer Algorithm. Sensors.

[22] An Q., Zhang J., An Q., Shamekov A. (2020). Quasi-Proportional-Resonant Controller Based Adaptive Position Observer for Sensorless Control of PMSM Drives Under Low Carrier Ratio. IEEE Trans. Ind. Electron..

[23] Wang G., Li Z., Zhang G., Yu Y., Xu D. (2012). Quadrature PLL-based high-order sliding-mode observer for IPMSM sensorless control with online MTPA control strategy. IEEE Trans. Energy Convers..

[24] Lara J., Xu J., Chandra A. (2016). A novel algorithm based on polynomial approximations for an efficient error compensation of magnetic analog encoders in PMSMs for EVs. IEEE Trans. Ind. Electron..

[25] Wang G., Li T., Zhang G., Gui X., Xu D. (2013). Position estimation error reduction using recursive-least-square adaptive filter for model-based sensorless interior permanent-magnet synchronous motor drives. IEEE Trans. Ind. Electron..

[26] Volpato Filho C.J., Vieira R.P. (2020). Adaptive full-order observer analysis and design for sensorless interior permanent magnet synchronous motors drives. IEEE Trans. Ind. Electron..

[27] Bolognani S., Calligaro S., Petrella R. (2014). Design issues and estimation errors analysis of back-EMF-based position and speed observer for SPM synchronous motors. IEEE J. Emerg. Sel. Top. Power Electron..

[28] Yang S.C., Chen G.R. (2017). High-speed position-sensorless drive of permanent-magnet machine using discrete-time EMF estimation. IEEE Trans. Ind. Electron..

[29] Harnefors L., Nee H.P. (2000). A general algorithm for speed and position estimation of AC motors. IEEE Trans. Ind. Electron..

[30] Zhang G., Wang G., Xu D., Zhao N. (2015). ADALINE-network-based PLL for position sensorless interior permanent magnet synchronous motor drives. IEEE Trans. Power Electron..

[31] Zou C., Liu B., Duan S., Li R. (2014). Stationary frame equivalent model of proportional-integral controller in dq synchronous frame. IEEE Trans. Power Electron..

[32] Yepes A.G., Freijedo F.D., Doval-Gandoy J., López Ó., Malvar J., Fernandez-Comesana P. (2010). Effects of discretization methods on the performance of resonant controllers. IEEE Trans. Power Electron..

[33] Bojoi R., Limongi L.R., Profumo F., Roiu D., Tenconi A. (2009). Analysis of current controllers for active power filters using selective harmonic compensation schemes. IEEJ Trans. Electr. Electron. Eng..

[34] Bojoi R.I., Griva G., Bostan V., Guerriero M., Farina F., Profumo F. (2005). Current control strategy for power conditioners using sinusoidal signal integrators in synchronous reference frame. IEEE Trans. Power Electron..

[35] Yepes A.G., Freijedo F.D., López O., Doval-Gandoy J. (2010). High-performance digital resonant controllers implemented with two integrators. IEEE Trans. Power Electron..

[36] Novak Z., Novak M. (2022). Adaptive PLL-Based Sensorless Control for Improved Dynamics of High-Speed PMSM. IEEE Trans. Power Electron..

[37] Raja R., Sebastian T., Wang M., Gebregergis A., Islam M.S. (2017). Effect of position sensor error on the performance of permanent magnet machine drives. IEEE Trans. Ind. Appl..

